# Virtual Partner Interaction (VPI): Exploring Novel Behaviors via Coordination Dynamics

**DOI:** 10.1371/journal.pone.0005749

**Published:** 2009-06-03

**Authors:** J. A. Scott Kelso, Gonzalo C. de Guzman, Colin Reveley, Emmanuelle Tognoli

**Affiliations:** 1 Human Brain and Behavior Laboratory, Center for Complex Systems and Brain Sciences, Florida Atlantic University, Boca Raton, Florida, United States of America; 2 Intelligent Systems Research Centre, University of Ulster, Derry, Northern Ireland; Indiana University, United States of America

## Abstract

Inspired by the dynamic clamp of cellular neuroscience, this paper introduces VPI—Virtual Partner Interaction—a coupled dynamical system for studying real time interaction between a human and a machine. In this proof of concept study, human subjects coordinate hand movements with a virtual partner, an avatar of a hand whose movements are driven by a computerized version of the Haken-Kelso-Bunz (HKB) equations that have been shown to govern basic forms of human coordination. As a surrogate system for human social coordination, VPI allows one to examine regions of the parameter space not typically explored during live interactions. A number of novel behaviors never previously observed are uncovered and accounted for. Having its basis in an empirically derived theory of human coordination, VPI offers a principled approach to human-machine interaction and opens up new ways to understand how humans interact with human-like machines including identification of underlying neural mechanisms.

## Introduction

In this paper we take inspiration from the “dynamic clamp” of cellular and computational neuroscience in order to probe essential properties of human social coordination. We do this by reciprocally coupling human subjects to a computationally implemented model of themselves, an invention we call Virtual Partner Interaction or VPI for short. In neuroscience, a dynamic clamp is an electrophysiological method that interfaces living cells dynamically to their simulated counterpart in order to explore cellular processes such as membrane or synaptic current transport. In one of its implementations, a circuit injects currents to a live neuron through a microelectrode inserted into its soma, simulating a synaptic process [1]. Output from the circuit is determined by a set of differential equations that constitute a computational model of neuronal behavior. Circuit input includes state-variables of the live neuron. A simulated neuron and a real neuron are therefore reciprocally coupled in real-time. This type of coupling between live and model neuron is called a ‘hybrid network’ [2], and acts as a bridge between experimental studies and computer modeling of neural networks. Properties of the interaction can be fully established by varying model parameters. Among its successes, the dynamic clamp has yielded insights into the role of voltage-dependent conductances and the timing of synaptic inputs (see [3] for a review). The motivation for the use of hybrid networks is to understand the consequences of the nonlinearities central to most physiological processes [2]. Often this involves studying the conditions required for different kinds of phase synchrony between cells [4]–[7].

**Figure 1:**
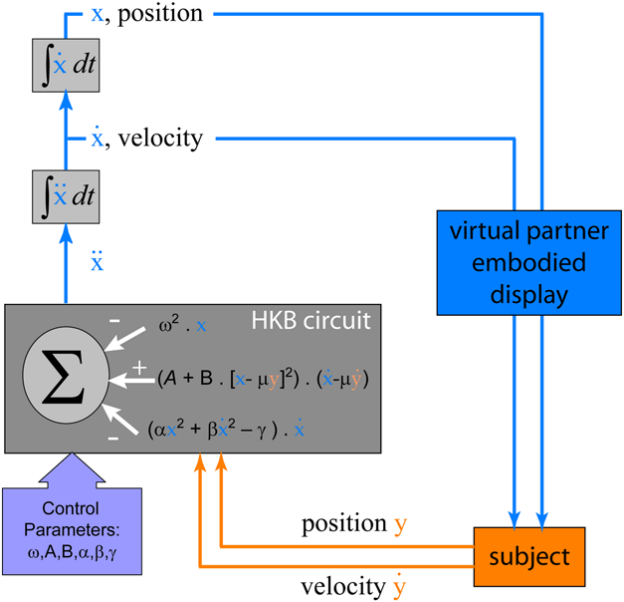
The Virtual Partner Interaction (VPI) paradigm. Subject coordinates finger movement with a virtual partner visually via an animated display. Subject's behavior 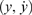 is digitized and fed to a real-time HKB computational circuit. The circuit computes corresponding virtual partner position and velocity 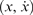 which is then used to animate the hand of the virtual partner. Circuit is coupled to the subject via the digitized inputs. Subject is coupled to the circuit visually via the display.

In like fashion, but now scaled up from the level of neuronal behavior to the level of behaving humans, we introduce VPI as a surrogate system to systematically investigate the essentially nonlinear dynamics of human social coordination (see [8]–[10] for recent reviews]. In VPI, a human being coordinates behavior with a virtual partner (sometimes referred to simply as VP in this paper) whose motion is driven by a nonlinearly coupled component oscillator of the Haken-Kelso-Bunz (HKB) model of coordination dynamics [11]–[14] the parameters of which depend on input from the human's own movements (Fig. 1). Coordinated movements between the human and the VP can vary from simple and repetitive to complex and discrete. They can be symmetrical or asymmetrical (both partners performing the same action or not), thereby laying the basis for such important behaviors as imitation learning or joint action with a shared goal. Basic coordination behaviors may be modeled using HKB dynamics. The HKB equations describe rhythmic coordination between similar effectors within as well as between individuals whose movements may be coupled through proprioception, vision or audition. The many extensions of the basic HKB equations are suitable for behaviors of further complexity. In the current implementation of VPI, the behaviors of both human and VP are chosen as rhythmic cycles of flexion and extension of the right index finger. The frequency and amplitude of the animated finger are determined by a real-time numerical simulation of the oscillator equation. The human subject's finger position and velocity are used to form the HKB coupling term for the oscillator, so that it reacts to the performance of the subject. The subject is visually coupled to the oscillator via the display so that the coupling is bi-directional.

The present approach is applicable to a wide range of human-machine interactions, in particular, to human-humanoid robots and their extension to multiple partner situations. Conceptual and technological advances have opened up many ways to explore and understand computational properties of neurobiological systems [15] as well as complex human-machine interactions [16]–[18]. Understanding these interactions is guided by models of information exchange characteristic of human social interaction [19]–[21]. In recent times, a shift has occurred toward creating humanoid machines that attempt to mimic human beings [22], be it for surrogate human interactions [21], [23]–[26], “intuitive or natural” human computer interactions [27], [28] or more broadly cognitive and behavioral cooperation between humans and machines [27], [29]–[35], including in rehabilitation settings [36]–[39]. Two main design themes or directions have emerged. The first attempts to build integrated architectures of functional systems, e.g. for perception, attention, spatial navigation, learning, decision-making and so forth. The second, guided by principles of phylogeny and ontogeny, attempts to self-organize basic building blocks into purposeful systems ([33], [40]–[42]; see also [43] for a similar goal at the neural level). Ever greater recognition is being given to the importance of coordination between the agent's “brain” and “body” [27], [33], [34], [41], [44], as well as to the social significance of behavior [33], [44], [45]. For individuals, behavior is a means of seeking energetic and informational resources in the environment [46]. Behavior (actions, gestures, facial expressions, verbal communication and so forth) also provides a means to integrate information about the self and the other, thereby supporting purposeful interactions. Adding to the significance of behavior for the emergence of social complexity, advanced brains appear to have evolved a specialized neural system for this function called the mirror neuron system [47]. The mirror neuron system has been assigned explanatory duty for numerous cognitive and social functions including theory of mind, language, empathy, cooperation and skill learning [48].

In VPI, the virtual partner is endowed with a coordination dynamics that is intended to capture how one human being performs visual coordination with another. First published in 1985, the HKB model of this coupled behavior is one of the most extensively tested quantitative models in human movement [49]. In its original form, HKB describes and predicts the dynamics (multistability, instability, transitions, etc) of the relative phase between two oscillating fingers or limbs when frequency or rate is scaled [50], [51]. In HKB, the equation of motion for the key collective or coordination variable (relative phase) can be derived by treating the interacting components as nonlinearly coupled nonlinear oscillators [11]. Much work has gone in to identifying the intrinsic properties of the components and their coupling (see [52] for a review). HKB has been successfully extended in numerous ways, for instance, to situations where different limbs are coordinated, movements are coordinated with different sensory modalities, multifrequency coordination as in drumming and piano playing, discrete as well as rhythmical movements—to name just a few. When combined with noninvasive brain imaging techniques, the HKB model (and more generally, the theoretical concepts and methods of coordination dynamics) have motivated new ways to investigate brain function (e.g. [53]–[59]).

One remarkable extension of HKB is that it describes and predicts basic patterns of social coordination between two people [60], [61]. It naturally follows that the HKB equations are suitable to design a ‘dynamic clamp’ for human-machine interactions that is modeled after human-human interactions. Analogous to the dynamic clamp [1], [3] VPI allows the experimenter to explore a range of control parameters and coupling manipulations not typically accessible in experimental studies of human social coordination. As proof of concept, we asked human subjects to coordinate rhythmic finger movements with a virtual partner and maintain in-phase coordination with the VP's movements. However, the virtual partner was parameterized to couple most stably *anti-phase* with its human counterpart. The outcome of pitting one behavior against the other, we hypothesized, is virtually guaranteed to be an emergent behavior that is dependent on neither the virtual partner nor the human subject alone, but rather to the cooperation or competition between them. As we will show, the experiment reveals phenomena consistent with the HKB model as well as a number of new effects (‘strategies’) never previously observed or anticipated in experimental studies of social coordination, but that are nevertheless understandable on further analysis.

## Materials and Methods

### The VPI system

A component equation of the HKB model is given by the non-linearly coupled nonlinear oscillator
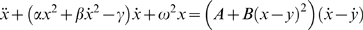
where *x* and *y* refer to the positions of two interacting partners (Fig. 1) and the parameters *α*, *β*, *γ*, *ω*, *A* and *B* are constants [11] (see Table 1). The equation for the second component *y* is obtained by a simple substitution *x→y*, *y→x* giving us a symmetric system. These equations are often simplified into phase and amplitude components which yield, under the rotating wave and slowly varying amplitude approximations [11], a relative phase dynamics that describes coordinated behavior between the interacting components (e.g., fingers, limb segments) within or between individuals. For the parameters used in [11], coordination at in-phase is more stable compared to anti-phase for frequencies comparable to those used in our VPI study (1 Hz to 3 Hz). In this paper, *x(t)* drives the movement of the virtual partner while the input *y(t)* is the actual movement from a human subject. A computer generated virtual partner (an avatar of a hand) is constructed using an animated sequence of index finger movements whose position is selected based on a mapping from the variable *x*. The human subject is visually coupled to the virtual partner through the animated display (Fig. 1). The oscillator is coupled to the human partner's motion *y(t)* via the modified coupling function 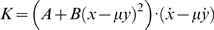. The parameter *μ* serves to scale the response of the human's movements to the dynamic range of the virtual partner's and to control for the virtual partner's preference for in-phase or anti-phase coordination with the subject. We used reversed coupling (*μ*<0) so that the virtual partner was parameterized to couple most stably anti-phase with the human subject creating, as it were, a “conflict of intentions”. The choice of oscillator and coupling parameters (Table 1) was guided by empirically obtained values fitted to a self-excited oscillator model of finger movements [11], [62] and the requirement that the VPI system produce an emergent behavior.

**Table 1:** Virtual Partner Interaction experiment parameters.

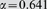
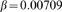
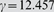
A = 0.12
B = 0.025

### Preliminary Simulations and Predictions

**Figure 2:**
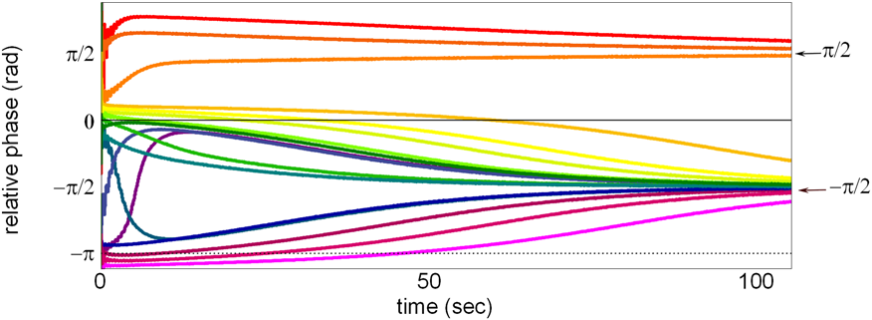
Simulation of the relative phase behavior of a reverse-coupled HKB system. Relative phase approaches either −π/2 or π/2 depending on the initial condition. Except for the reversed coupling, the parameters used are identical and are given in Table I. The shifted attractors are reminiscent of the bi-stability at 0 and π found in the normally coupled HKB system. The convergence of the trajectories toward two attractors at −π/2 and π/2 reflects the (minimal) bistability present due to the choice of parameters.

**Figure 3:**
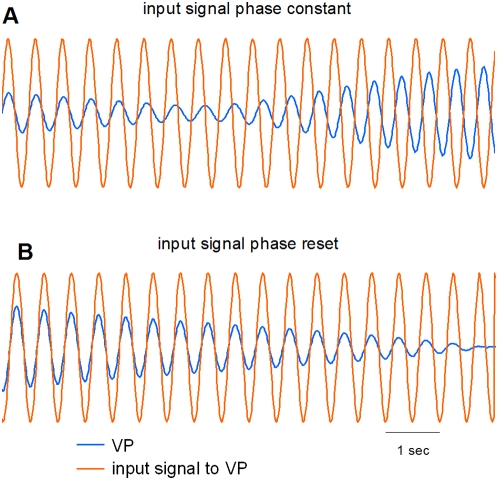
Response of the virtual partner (blue curve) to a sinusoidal input (orange curve). Sine input has the same frequency and fixed amplitude. The plots are time series of positions. (A) After starting out at in-phase, the coordination pattern switches to the virtual partner's preference at anti-phase. This switch is accompanied first by reduction then by an increase in the amplitude. (B) If the sinusoidal input is periodically reset so as to be in-phase with the virtual partner, the virtual partner amplitude decreases and does not recover. For the full VPI experiment, this has the effect of degrading the visual information required by the subject to coordinate effectively with the virtual partner.

In Fig. 2 are shown examples of the relative phase behaviors one expects for a reversely coupled (*μ* = −1) HKB system under various random initial conditions but with otherwise identical oscillator and coupling parameters (Table 1). Instead of settling down to attractors at *ϕ* = 0 and *ϕ = π* (in-phase and anti-phase) as is the case for the normally coupled HKB system with *μ* = 1, the relative phases now approach the intermediate values π/2 or −π/2 depending on their initial condition. Note that the relative phase attractors at π/2 and −π/2 in the reverse coupled case, though rare, are also approximately achieved in studies of spontaneous coordination between two people but the nature of this behavior (e.g. intention, reversed coupling) is not known at the moment. What is known is that it is more common in behavioral coordination studies involving nearly identical frequencies (whether between effectors, between two people, or between a subject and an external stimulus) to have in-phase and anti-phase as stable patterns. Thus, an interesting question here is which relative phase patterns actually emerge under the experimental conditions of VPI. We also explored the virtual partner's response to a synthesized sine signal, 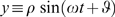, whose frequency *ω*, amplitude *ρ*, and phase angle *ϕ* can be varied at will (Fig. 3). This input to the virtual partner does not have the intrinsic dynamics (e.g. self-excitation, amplitude-frequency relation) of an HKB component, but it can serve as an aid in constraining the parameters of the full VPI experiment. Switching is of particular interest since it usually provides the most information about mechanisms underlying behavior in a dynamical system. For the results shown in Fig. 3, we used the parameters in Table 1 that were employed in the full VPI experiment. In Fig. 3a, we show a behavior similar to the characteristic amplitude decrease/increase in component oscillations in the HKB-model when there is a switch in coordination pattern. However, since the avatar is reverse-coupled to the sinusoidal input, we see a switch from in-phase to anti-phase. In Fig. 3b, instead of allowing the coordination pattern to switch to anti-phase, the phase of the sinusoidal input is reset so that it is always in-phase with the virtual partner. This has the consequence that the VP amplitude does not recover. The foregoing simulations suggest that subjects who persistently coordinate in-phase with the virtual partner will eventually encounter difficulty in perceiving the avatar's behavior because of the degradation in oscillator amplitude. How subjects solve this problem is explored in the experiment.

### The Experiment

#### Ethics Statement and Subjects

Ten subjects (6 female and 4 male; 18 to 35 years old) provided written informed consent prior to the experiment and were included in the study. Procedures were approved by the Internal Review Board at Florida Atlantic University and conformed to the principles expressed in the Declaration of Helsinki. All subjects were right handed and had normal or corrected-to-normal vision.

#### Task

The experiment consisted of two initial scaling trials and 32 experimental trials. Scaling trials lasted 200 sec., and experimental trials lasted 100 sec. Subjects were instructed to maintain smooth, rhythmic movements with the right index finger (flexion-extension) and to avoid stopping their finger at any time. Since the frequencies in the experiment were low, fatigue was not a factor.

**Figure 4:**
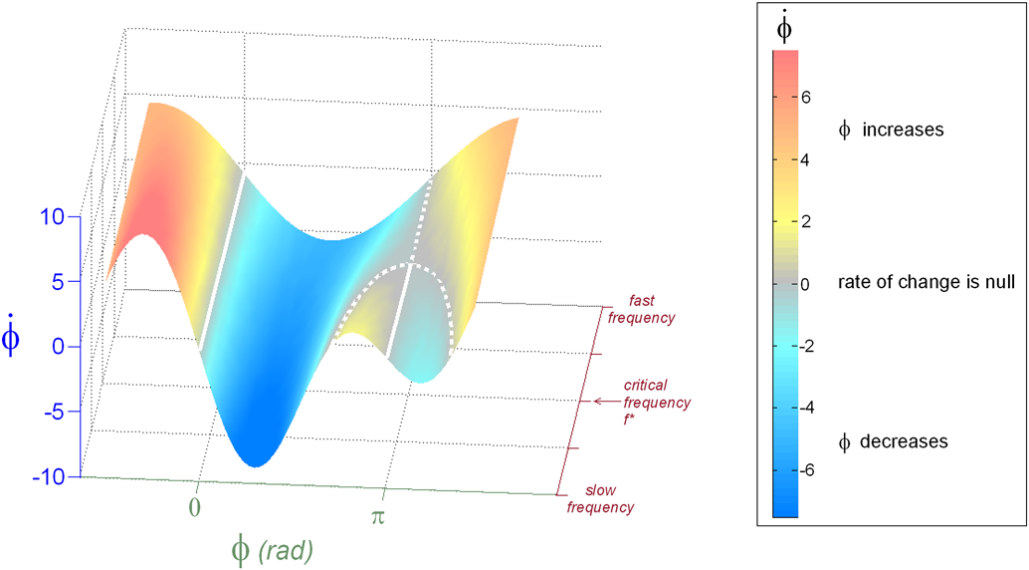
Selection of experimental frequencies guided by the HKB collective variable dynamics. Humans have shown remarkably consistent coordinative (relative phase) behavior in a wide variety of coordination tasks with rhythmic stimuli, a fact captured by the elementary HKB dynamics [11] illustrated here. When asked to synchronize at the same frequency with the stimulus, stable phase patterns are invariably present at (or close to) anti-phase and in-phase for low movement frequencies (typically <2 Hz). This is indicated by the solid lines of fixed points (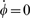) when 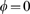 and 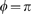 for *f* below a critical frequency f*. For frequencies *f*>f*, only the fixed point at 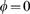 is stable. In the VPI experiment, a separate scaling trial in which the frequency is systematically increased is used to determine f*. The value of f* is then used as an upper bound for the choice of frequency parameter, ensuring that pattern instability is not only due to the effect of high frequency in the subject but also comes from conflicting tasks.

The scaling trials determine the average critical movement frequency 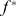 (in Hz) at which a subject loses anti-phase coordination with a (moving hand) visual stimulus as the frequency of the stimulus is increased from 1.5 Hz to 3.3 Hz in increments of 0.2 Hz every 20 seconds [14]. Figure 4 illustrates the key points in the scaling trial. This frequency is used to determine low 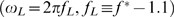 and high 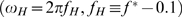 oscillator frequency parameters (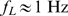 and 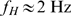). Both *f_L_* and *f_H_* frequencies belong to bistable regimes in which the human can sustain in-phase and anti-phase coordination, i.e. realizations of both human and VP “intentions” are possible. The faster frequency was employed because it tends to promote more intermittent switching behavior: coordinating at faster rates enhances fluctuations, thereby creating opportunities for the partners to switch between states.

**Figure 5:**
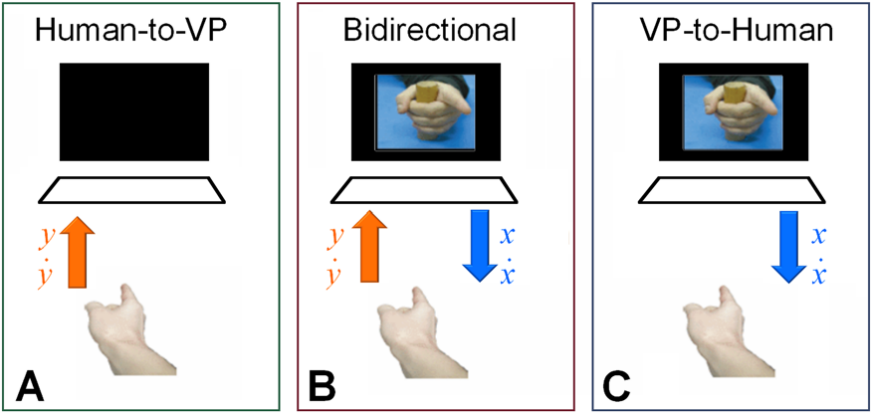
Experimental conditions defined by the direction of coupling or information flow. In human-to-VP condition (A), the display is switched off but kinematic information about the subject's movement is received by the virtual partner. In the bidirectional condition (B), the subject sees the virtual partner's movements and the virtual partner receives kinematic information of the subject's movements. In the VP-to-human condition (C), a subject has vision of the virtual partner's movements but the virtual partner is decoupled (coupling term set to zero) from the subject.

Based on the foregoing considerations, the main experimental design consisted of two rates (low and high frequency) ×3 conditions (one bidirectional and two unidirectional coupling conditions). We label these conditions in terms of information flow between the human and the virtual partner, i.e. who affects whom (Fig. 5). For the bidirectional coupling condition, the partners are reciprocally coupled: information flows to the VP through the coupling term of the HKB equation and to the subject through vision of the animated display. In the human-to-VP condition, the oscillator receives kinematic information about the human's behavior which is processed through the HKB coupling term, but the animated display is switched off, so that the human is decoupled from the oscillator. In the VP-to-human condition the human sees the animation displayed, but the coupling term of the oscillator is set to 0, so that VP motion is intrinsic and independent of the human's behavior: the VP acts essentially like a metronome [14]. The purpose of the VP-to-human trials is to check that we are in a region of parameter space that promotes bidirectional interaction and not simply coordinating with a metronomic stimulus disguised as an avatar. Similarly, the purpose of the human-to-VP trials is to ensure that the oscillator itself is not capable of inducing phenomena of note without the presence of human interaction. Subjects were paced for 5 sec. prior to trial onset to entrain them to a movement frequency that was identical to the virtual partner's (*f_L_* or *f_H_*). In both the bidirectional and VP-to-human conditions, subjects were asked to coordinate finger movement in-phase with the virtual hand. In the human-to-VP condition, subjects were asked to maintain a continuous movement for the duration of the trial. Trials (16 bidirectional, 8 human-to-VP, 8 VP-to-human; half of which were at low and high frequency respectively) were presented in a random order.

#### Apparatus

The position and velocity of the subject's index finger was measured via a manipulandum that rotated freely in the transverse (horizontal) plane about a fixed axis aligned with the metacarpophalangeal joint. Position data (angular displacement), measured by a DC potentiometer, were acquired at a sampling rate of 1 KHz using a National Instruments A/D converter and down-sampled by the computer program to 100 Hz. Velocity was numerically computed using a 3-point differentiation algorithm and, together with the position data, used to form the coupling term with the HKB oscillator. The position of the oscillator was used to select one of 119 position-indexed images, which were displayed on the screen. The screen animation was refreshed at 100 Hz during the experiment and looked just like a normal video (Fig. 5).

#### Analysis

Raw data from the subjects' movements were pre-processed by application of a digital low pass filter (Butterworth, 10 Hz, recursively applied for zero phase-shift). Frequency was estimated via a wavelet transform (Morlet mother wavelet). Relative phase of the subject with respect to the virtual partner was computed using a continuous Hilbert transform on the mean-centered position data. Collective behavior was classified as stable, switching, and unstable according to a combined measure of synchronization index (*SI*) [63], [64] and dwell time *τ* around regions near in-phase. The dwell time relates to sustainability of the coordination pattern over the longer time scale (on the order of the trial length), whereas the synchronization index is also sensitive to stabilization of the relative phase at the shorter time scale (on the order of a movement cycle length). *SI* is based on the circular variance (*CV*) of the relative phase over the whole trial and is defined as
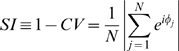
where *φ_j_* is the relative phase at time *t_j_ = jΔt*, where *Δt* is the sampling interval. Total dwell time is defined as *τ = Σ τ_n_*, where *τ_n_* is the local dwell time for the *n^th^* phase-locked interval within the trial and the summation is taken over all such intervals. Episodes of coordination either span the entire trial or are established and lost recurrently over its course. Each episode of stabilization of the relative phase that lasts more than 2 cycles with a variation about the mean of less than 0.17 radians (15 degrees) is called a local dwell time *τ_n_*. Trials were classified as stable if they exhibited extended phase locked intervals and had *SI*>0.8 and a single dwell time *τ*≥90% of the trial duration. Switching trials were classified as such if they showed transitions from in-phase and back and had 0.3≤*SI*≤0.8 with a *cumulative* dwell time *τ*≥25%. Unstable trials have *SI*<0.3 and show characteristic phase wrapping throughout most of the trial.

## Results

**Figure 6:**
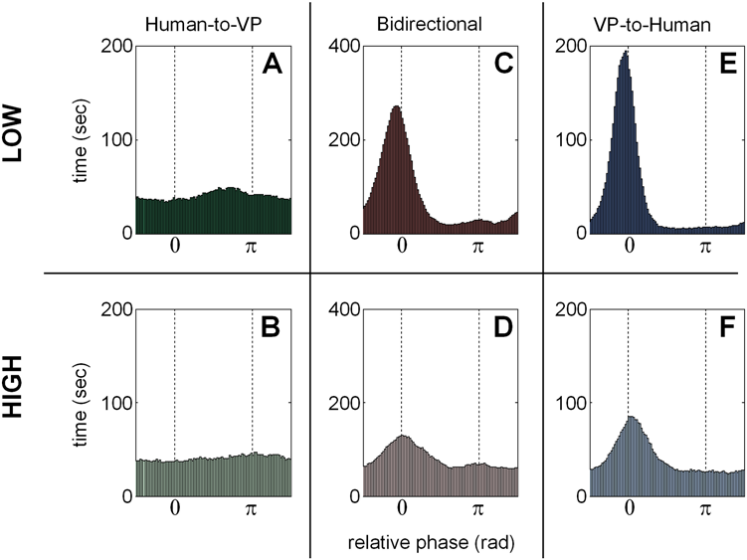
Relative phase distributions for unidirectional and bidirectional conditions at low and high movement frequencies. Data are collapsed across subjects. For the Human-to-VP conditions, the distributions of relative phase suggest peaks at just below anti-phase (≈2.5 rad) for the low-frequency condition (A) and near anti-phase (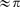 rad) for the high-frequency condition (B). The relatively flat distribution shows the weakness of the coupling of the virtual partner with the human. On the other hand, in the VP-to-Human conditions (E) and (F) the human subject is able to coordinate with the virtual partner when the latter functions like a passive visual metronome. The results for Bidirectional conditions are shown for low (C) and high (D) frequencies, respectively. The range of the vertical axis is doubled compared to unidirectional conditions because of the different number of trials used. The distributions are bimodal with a larger concentration of in-phase than anti-phase at both frequencies. For high (D) relative to low frequency (C) the concentration at in-phase decreases while phase dispersion and antiphase increase.

First we present the relative phase distributions from the unidirectional and bidirectional conditions. This comparison is to verify that we are in a parameter region where the coupled behavior is truly reciprocal. In Fig. 6 we plot the relative phase distributions for both unidirectional and bidirectional coupling conditions, each collapsed across all subjects and trials. The distributions of the relative phase in human-to-VP conditions (Figs. 6a,b) show the weakness of the coupling of the virtual partner with the human. A faint peak is observed just below anti-phase (≈2.5 rad) for the low-frequency condition (Fig. 6a) and near anti-phase (≈π rad) for the high-frequency condition (Fig. 6b). On the other hand Figs. 6e,f indicate that the human subject is able to achieve synchronization with the avatar acting as a non-interacting partner (i.e., as a visual metronome). Here, in both low and high frequency conditions, a marked peak in the relative phase is observed at in-phase. In the bidirectional coupling trials (Figs. 6c,d), a major relative phase concentration is observed at in-phase as in the previous case, but there also emerges a minor relative phase concentration at anti-phase. Despite the weakness of the virtual partner's coupling with the human, the virtual partner can induce the human toward its preferred coordination behavior as well as being influenced by him/her.

**Figure 7:**
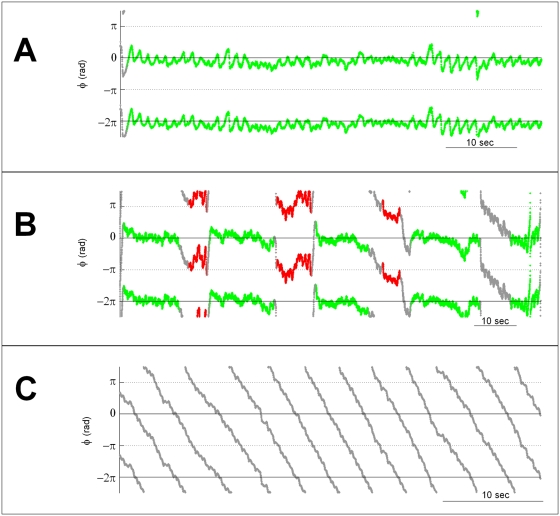
Examples of relative phase time series showing the three basic behaviors found in bidirectional trials. Stable coordination is shown in (A), intermittent switching between in-phase and anti-phase in (B) and unstable phase wrapping behavior in (C). Using the synchronization index and dwell time criteria, the percentage distributions were computed and are given in Table 2 (for comparison, data for the unidirectional conditions are also provided).

Although comparisons between the gross distributions in Fig. 6 are indicative of emergent behavior, a clearer picture of interaction is obtained through analysis of the basic time series. Figure 7 shows relative phase time series of the three basic behaviors found in bidirectional trials: stable, switching, and unstable. Using synchronization index and dwell time criteria, the percentage distributions were computed and are presented in Table 2. For comparison purposes, data for the unidirectional conditions are also provided. From Table 2 we see that as in reciprocally coupled live interactions between two people, movement rate determines the stability of coordination [60], [61], [65]. When the task for the subject was to coordinate with the virtual partner at low frequency, 42.5% of the trials were stable, 37.5% exhibited switching, and 20% were unstable. At high frequencies, only 2.5% of the trials were stable, 32.5% exhibited switching, and 65% were unstable. The low percentage of stable trials at higher frequencies was predicted on both empirical and theoretical grounds. Near 2 Hz, in the absence of special training, subjects start to lose synchronization even with a passive visual metronome [14]. In the presence of an opposing partner that seeks anti-phase (as is the case with the VP here), it was expected that loss of coordination around 2 Hz may be even more prevalent.

**Table 2:** Distribution of coordination patterns for low and high frequency conditions classified according to combined criteria of synchronization index and dwell time.

Pattern	Human-to-VP	Bidirectional	VP-to-Human
Stable	0% (low)	42.5% (low)	75.0% (low)
	0% (high)	2.5% (high)	2.5% (high)
Switching	2.5% (low)	37.5% (low)	12.5% (low)
	2.5% (high)	32.5% (high)	42.5% (high)
Unstable	97.5% (low)	20.0% (low)	12.5% (low)
	97.5% (high)	65.0% (high)	55.0% (high)

**Figure 8:**
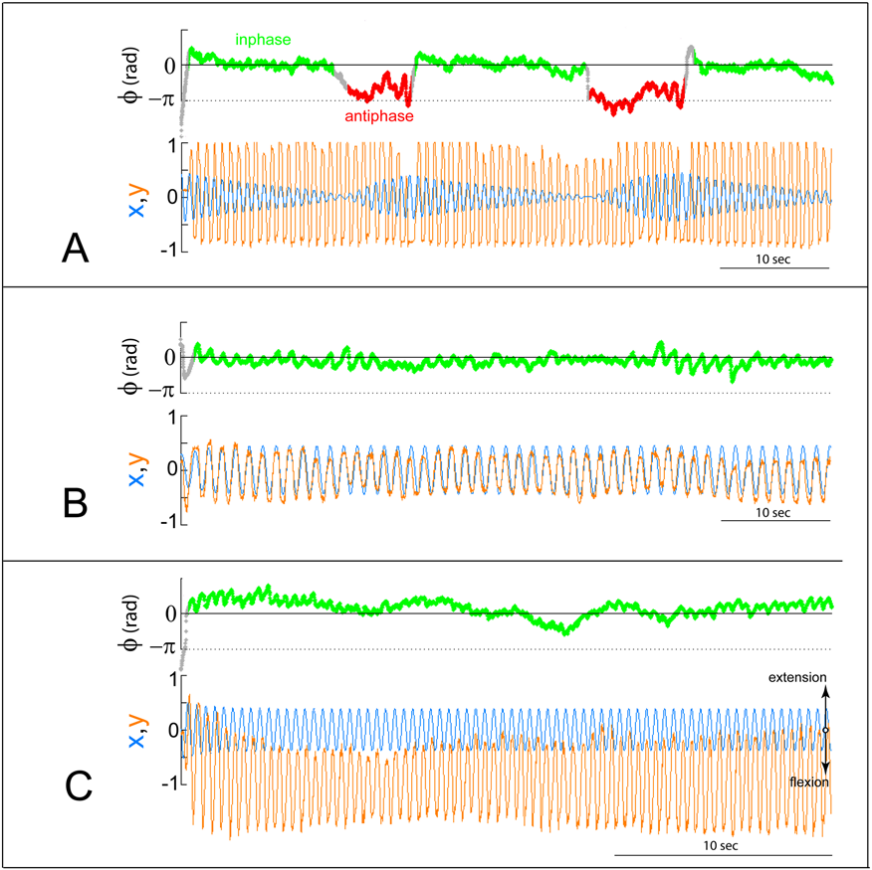
Behavioral patterns in bidirectional conditions. Reciprocal interaction between human and VP gives rise to unstable (not shown), intermittent (A) and stable (B, C) collective behaviors. Shown are the time series for positions of the virtual partner (x, blue curve) and the subject (y, orange curve) and the relative phase 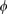 of the subject with respect to the virtual partner. Motion near in-phase and anti-phase are highlighted in green and red, respectively. When a subject is in-phase with the virtual partner, the latter's amplitude eventually decreases due to the reversed coupling. To prevent amplitude collapse, subjects may temporarily switch to anti-phase (A). For extended in-phase coordination, spatial strategies were employed by the subjects. These include reducing one's amplitude to an optimal range (B), and shifting the center of oscillation downward toward flexion (C). None of the strategies were part of the instructions to coordinate but were discovered during the course of interaction.

In addition to the usual effects seen in coordination studies [11]–[14], [50], [51], [60], [61], [65], novel and unanticipated behaviors were uncovered. Due to the reversed HKB coupling built into the virtual partner, extended in-phase coordination with the human subject (especially at low frequency) depresses the movement amplitude of the virtual partner thereby degrading the visual information required for accurate coordination. The amplitude drop is consistent with our analyses of the oscillator's response to a synthesized sine signal whose phase is reset to enforce prolonged synchronization at in-phase (Fig. 3b). Thus, the human subject not only has to keep pace with the virtual partner, but also has to sustain the virtual partner's amplitude of motion. To coordinate effectively, successful subjects adopted several “strategies”. We use the word “strategy” here only as way to categorize the behaviors produced. There is no indication that these behaviors were planned or decided in advance and every indication that they emerged in real time as a result of the particular experimental circumstances. In the first strategy, subjects induced amplitude recovery in the virtual partner by switching temporarily to anti-phase (Fig. 8a). Each switch brings up the virtual partner's amplitude long enough to accomplish synchronization. Thus, subjects maximize time in the instructed in phase pattern by allowing brief incursions of anti-phase coordination during which the VP satisfies its own requirement.

In a second strategy, subjects adjusted the spatial properties of their movements by either maintaining their amplitude within an optimal range (Fig. 8b) or shifting their center of oscillation toward the direction of flexion (Fig. 8c). It is important to emphasize that these novel ‘strategies’ were not part of the instructions but were discovered during the course of the interaction (see also footnote 1). On exit interviews, some subjects even reported that the machine was “messing” with them, suggesting the attribution of agency or intentional state to the virtual partner.

**Figure 9:**
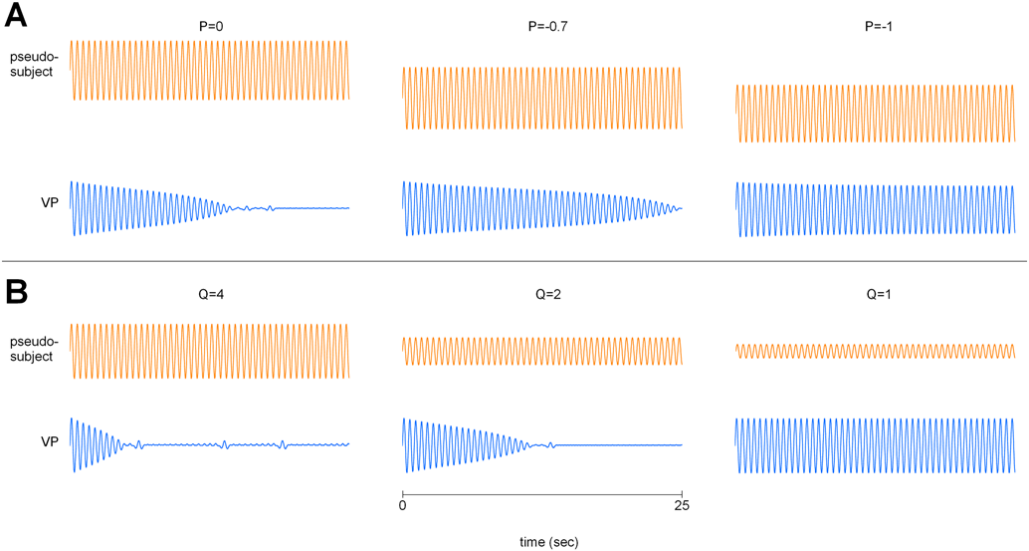
Simulations of spatial strategies during extended in-phase coordination. A sine signal acts as a pseudo-subject for the virtual partner. The phase of the sine signal is reset to force in-phase synchronization. The plots show the position time series of the VP (blue) in response to amplitude and origin shift manipulations of the input signal (orange). (A) The amplitude decline of the VP is systematically delayed when the origin of the sine oscillation is changed by amounts P = 0, −0.7, and −1 (shifted down). At P = −1, the virtual partner's amplitude remains constant throughout the 100 sec simulated trial. (B) When the effective input amplitude Q is systematically reduced (Q = 4,2,1), the decline in the virtual partner's amplitude is also delayed. At the critical value Q = 1, the virtual partner maintains its amplitude throughout the run.

To understand how the spatial strategies (Figs. 8b,c) affect the motion of the virtual partner, it is enough to note the effect of a simple linear transformation of the subject's position input on the instantaneous oscillator-to-subject coupling. Thus, for an origin shift, *y→p+y*, the coupling is incremented by an amount 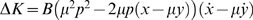. Likewise, a decrease or increase in subject amplitude by a factor *q* effectively changes the scaling from *μ* to *qμ* thereby also affecting the coupling. Both manipulations potentially impact the virtual partner, acting in effect as additional reverse damping mechanisms. As in the simulations shown in Fig. 3, we used a sine signal as an idealized ‘pseudo subject’ able to deliberately shift origin and change amplitude at will, and yet maintain in-phase coordination with the virtual partner. The results shown in Fig. 9 are presented in terms of measured outcomes rather than *p* and *q* because of conversion factors inherent in analog-to-digital systems. In Fig. 9a we used three decreasing *p* values resulting in the measured origin being successively shifted down by the amounts *P* = 0, *P* = −0.7, and *P* = −1.0. Note that the decline in the virtual partner's amplitude is also progressively delayed until *P* = −1.0 where it is sustained throughout the length of the simulation. In Fig. 9b, three decreasing values of *q* corresponding to measured subject amplitudes *Q* = 4, 2, and 1 were used. For *Q* = 1, the virtual partner's amplitude is effectively maintained. These modeling studies nicely capture the novel behaviors produced by subjects to preserve the amplitude of the VP.

## Discussion

VPI provides an attractive new frontier for human-machine interaction. Whereas artificial systems can be elaborated and theorized about, the human response (and consequently the coupled response) is less well-known. In this paper, the emphasis has been on examining the continuous dynamics of interaction between a human and a machine whose dynamics is similar to that of the human. The coupled dynamics is based on equations of motion that have successfully described coordinated behaviors within and between individuals, now extrapolated to hybrid settings (i.e., co-existence of human and computational agents interacting in real time). In the present work we have uncovered complex emergent behaviors under parsimonious experimental settings and discovered salient features of the interaction (here, coordination of rhythmic behaviors between two dynamically similar systems). This step complements the conventional input/output paradigm which may not always capture the complexity of interaction [66].

As reviewed in the introduction, outside of the present framework of coordination dynamics there have been many extensive studies of human-machine interaction covering a wide variety of contexts. One salient example in the spirit of VPI uses neural networks and game theory to simulate the “paper-rock-scissors” game [67] wherein individual players are pitted against their neural network counterpart. By manipulating parameters such as the amount of working memory and an operational measure of perceived outcomes, the game could be biased in favor of one player over the other, a result not predicted from game theory. More recently, again in a similar vein to VPI, Repp and Keller [68] have studied sensorimotor synchronization with a simulated partner whose output is based on an extended model of self-paced finger tapping.

There is much more to simple finger movements than meets the eye. A great benefit of the present approach is that the VP is based on detailed empirical studies and theoretical modeling of the component oscillator's dynamic features (at both behavioral and neural levels) as well as its fundamental biophysical coupling. Bearing in mind that the virtual partner alone cannot enforce anti-phase coordination (Figs. 6a,b), the observation that the coordination pattern may switch, if only temporarily, from in-phase to anti-phase (Figs. 7b and 8a) during reciprocal interactions is quite remarkable. In two person interactions the switch is typically from the relatively less stable anti-phase to the more stable in-phase pattern. Our data suggest that the virtual partner is not faithfully following the input of the human subject to effect an anti-phase pattern. More likely, the switch is induced on the human subject by the virtual partner. As for the spatial strategies that subjects use to overcome the amplitude reduction of the virtual partner, we note again that such actions were not part of the instructions on how to coordinate, but were discovered by the human subjects during the course of the interaction. Typically, analyses of coordination between two people have focused on the relative timing (relative phase) between important events such as peaks or troughs. Often, neither the amplitude nor the exact position of the center of oscillation has been extensively studied (but see [69]).

In typical social coordination experiments, [e.g. 60], [61], [65], the “parameters” of the behavioral dynamics expressed by the subjects may only be influenced by the experimenter indirectly (i.e. by instruction), and may even be in flux during the experiment as the intentions of each party are subject to change. Coupling a human to a model clamps the parameters of one of the parties, so that the task of identifying the properties of the other party is simplified: yet both parties remain dynamic in the sense that they react to and interact with each other. The interaction is richly reciprocal, in the same way that social coordination is reciprocal: Party A affects party B, and party B simultaneously affects party A. This may be contrasted to unidirectional interaction with a passive stimulus such as a metronome, in which only one party may be said to be “dynamic.”

Analogous to the dynamic clamp in cellular and computational neuroscience, VPI provides an opportunity to explore parameter ranges and perturbations that are not easily implemented in traditional live social interaction studies. This extended parameter range opens up the possibility of systematically driving neuromarkers –dynamical brain processes involved in social interaction [65]– to better understand their roles and may also lead to novel applications. For example, in modern society people have to deal with new technology that sometimes does not provide immediate “affordances”–qualities of an object that allow users to discover their function without the requirement of instruction or learning [70]. Interactions with ever proliferating technological devices often place high skill demands on users who have little time to develop those skills. The opportunity presented through VPI is that equally useful and informative new behaviors may be uncovered despite the built-in asymmetry in the human-machine interaction. Modifying the dynamics of the virtual partner with the purpose of inducing a desired human behavior (e.g. as in learning a new skill or as a tool for therapy and rehabilitation) is another useful possibility. On a more basic level, there is also a great deal of interest in engineering complex dynamic structures to produce desired states [71]. For example, weak nondestructive signals can be used to alter interactions among nonlinear rhythmic electro-chemical elements [72]. In a similar way, VPI brings the human into the picture: the human may tune the response of the machine and the machine may tune the response of the human. In principle, the VPI invention can be scaled up to include multiple partners and multiple sensory modalities. Frames of reference and mappings between human and machine can be explored. Equations of motion that have been proposed to handle discrete as well as rhythmic behaviors can be readily incorporated [73]–[75]. Indeed, it seems that VPI—due to its grounding in empirically-based models of coordination dynamics—opens up the possibility of exploring and understanding a wide variety of interactions between minds and machines.
